# STROKE34 Study Protocol: A Randomized Controlled Phase IIa Trial of Intra-Arterial CD34+ Cells in Acute Ischemic Stroke

**DOI:** 10.3389/fneur.2018.00302

**Published:** 2018-05-07

**Authors:** João Sargento-Freitas, Anabela Pereira, André Gomes, Paula Amorim, Teresa Matos, Carla M. P. Cardoso, Fernando Silva, Gustavo Cordeiro Santo, César Nunes, Orlando Galego, José Carda, João Branco, Víctor Lourenço, Luís Cunha, Lino Ferreira

**Affiliations:** ^1^Centro Hospitalar e Universitário de Coimbra, Coimbra, Portugal; ^2^Faculdade de Medicina da Universidade de Coimbra, Coimbra, Portugal; ^3^Centro de Medicina de Reabilitação do Centro – Rovisco Pais, Tocha, Portugal; ^4^Crioestaminal, Cantanhede, Portugal; ^5^Centro de Neurociências e Biologia Celular, Coimbra, Portugal

**Keywords:** clinical trial, intervention, ischemic stroke, magnetic resonance imaging, stem cells, stroke

## Abstract

**Rationale/aim:**

Despite the increasing efficacy of recanalization therapies for acute ischemic stroke, a large number of patients are left with long-term functional impairment, devoid of efficacious treatments. CD34+ cells comprise a subset of bone marrow-derived mononuclear cells with the capacity to promote angiogenesis in ischemic lesions and have shown promising results in observational and *in vitro* studies. In this study, we aim to assess the efficacy of an autotransplant of CD34+ cells in acute ischemic stroke.

**Sample size estimates:**

30 patients will be randomized for a power of 90% and alpha of 0.05 to detect a difference in 3 months infarct volume.

**Methods and design:**

We will screen 18–80 years old patients with acute ischemic stroke due to occlusion of a middle cerebral artery (MCA) for randomization. Persistent arterial occlusions, contra-indications to magnetic resonance imaging (MRI), premorbid dependency, or other severe diseases will be excluded. Treatment will involve bone marrow aspiration, selection of CD34+ cells, and their administration intra-arterially in the symptomatic MCA by angiography. Patients will be randomized for treatment at 7 (±2) days, 20 (±5 days) or sham procedure, 10 in each group.

**Study outcomes:**

The primary outcome will be infarct volume in MRI performed at 3 months. Secondary outcomes will include adverse events and multidimensional functional and neurological measures.

**Discussion/conclusion:**

STROKE34 is a PROBE design phase IIa clinical trial to assess the efficacy of intra-arterial administration of CD34+ cells 7 and 20 days after acute ischemic stroke.

**Trial registration (EU Clinical Trials Register):**

2017-002456-88.

## Introduction and Rationale

Current treatment algorithms for ischemic stroke have their primary focus on promoting vascular reperfusion, leaving tissue recovery devoid of directed and efficient therapies. In fact, despite the increasing efficacy of recanalization methods, up to half of patients who survive ischemic stroke develop long-term functional impairment. To overcome this therapeutic need, cell therapies have emerged as a potential method of promoting neurological recovery (1–5). However, many quandaries still limit their translation into clinical practice, especially in therapies aiming at neuro and synaptogenesis (6). Recently, CD34+ cells have shown promising results in observational and *in vitro* studies, urging research translation into randomized trials (7–9). They comprise a subset of bone marrow-derived mononuclear cells representing the main source of endogenous endothelial progenitor cells (EPC), with the capacity to promote angiogenesis in ischemic injuries. A phase I clinical trial demonstrated the safety and feasibility of administering intra-arterially autologous, bone marrow-derived CD34+ cells (10). We aim to design a phase IIa clinical trial to evaluate the efficacy of the intra-arterial administration of CD34+ at two timepoints: 7 ± 2 and 20 ± 5 days.

## Methods and Analysis

### Design

STROKE34 is a phase IIa superiority, unicenter, randomized, blinded outcome assessment, sham-controlled clinical trial evaluating the efficacy of intra-arterially administered CD34+ cells 7 ± 2 and 20 ± 5 days after ischemic stroke (PROBE design).

### Patient Population

We will screen for inclusion consecutive patients with non-lacunar acute ischemic stroke infarctions within the territory supplied by a middle cerebral artery (MCA). In Table 1, we present the study’s inclusion and exclusion criteria. Demonstration of arterial recanalization at the time of inclusion will be determined by angiography (modified TICI grades 2b or 3) or transcranial color coded Doppler (TIBI grades 4 or 5) (11, 12).

**Table 1:** Inclusion and exclusion criteria for the study.

Inclusion criteria	Exclusion criteria
Age 18–80 years; Acute hemispheric ischemic stroke attributable to injury within the territory supplied by the middle cerebral artery; Symptomatic arterial territory is recanalyzed; Onset of an acute ischemic stroke that can have full clinical, imagiological, and bone marrow collection within 7 days after onset. Onset is defined as the time that the subject was last seen in a normal state, or bedtime for unwitnessed strokes occurring during sleep; Readily accessible peripheral venous access blood sampling; Ability to understand the requirements of the study and be willing to provide written informed consent, as evidenced by signature on an informed consent document, agreeing to perform the required assessments. In the event of incapacitated subjects, informed consent will be sought from a legally acceptable representative.	Patients found delirious, comatose, demented or having any mental impairment other than the neurological deficits related to the index stroke that in the investigator’s opinion renders the subject incapable to participate in the study; Presence of high-grade (>70%) internal carotid artery stenosis or occlusion ipsilateral to the current stroke. Inflammatory disease present at baseline (chronic systemic inflammatory disease active at the time of inclusion or acute inflammatory disease such as an infection); Active malignancy, or recent surgery (within the previous 3 months); Premorbid neurological deficits and functional limitations assessed by a premorbid Modified Rankin Scale (mRS) score >2; Presence of a severe co-existing disease that may interfere with the conduct of the study, irrespective of stroke outcome; Known pregnancy. Females of childbearing potential will be screened at baseline with urine pregnancy test and positive results will be excluded [the choice of excluding pregnancies is due to the relative contra-indication to magnetic resonance imaging (MRI) in these patients]; Contra-indication to MRI. Allergy to contrast agents.

### Randomization

**Figure 1:**
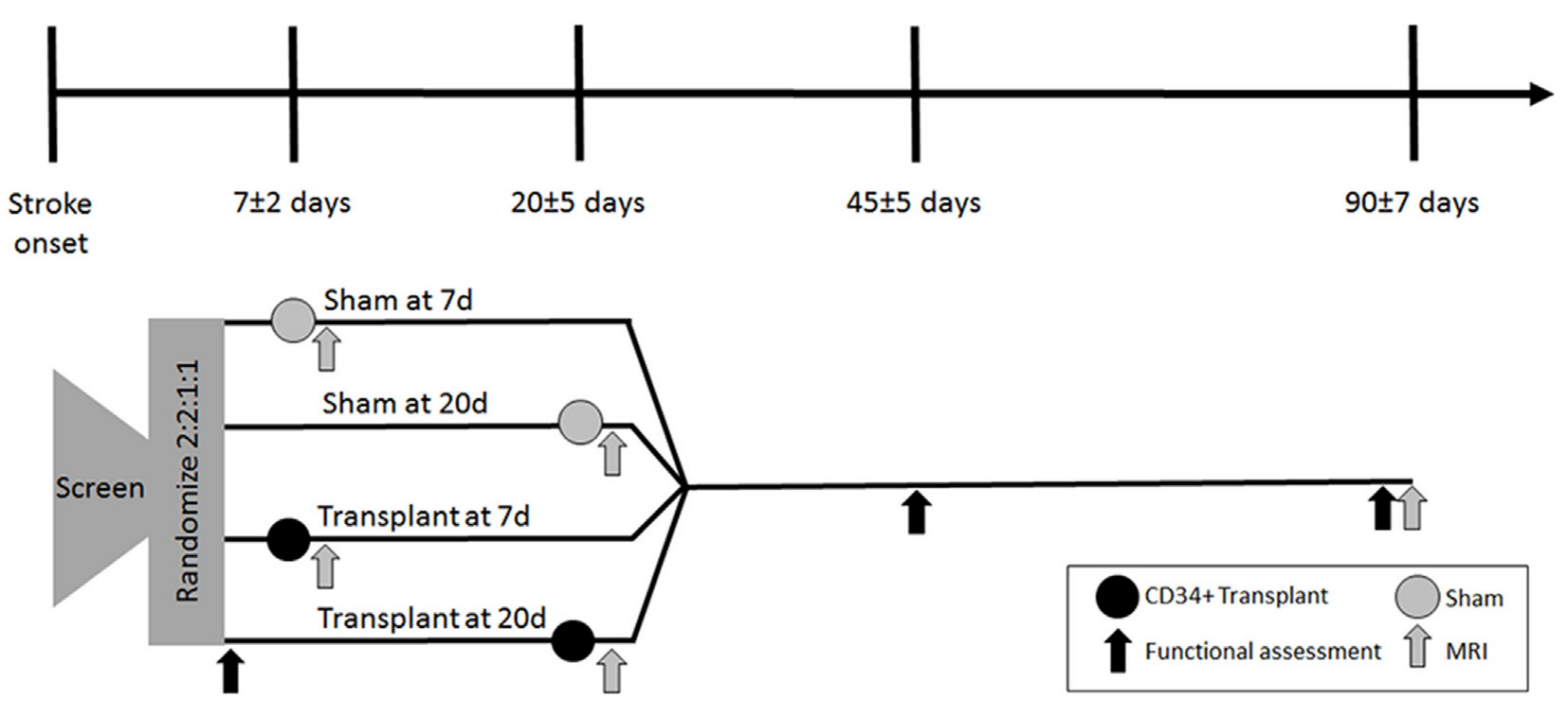
Trial design flow chart. Abbreviations: MRI, magnetic resonance imaging; d, days.

Subjects will be randomized, stratified for age, gender, baseline NIHSS (at randomization), laterality and performance of recanalization therapies (intravenous or intra-arterial) into intervention at day 7 ± 2; intervention at day 20 ± 5; sham procedure at day 7 ± 2; sham at day 20 ± 5, in a 2:2:1:1 ratio, using a computerized stratified randomization program (Figure 1).

### Intervention

**Figure 2:**
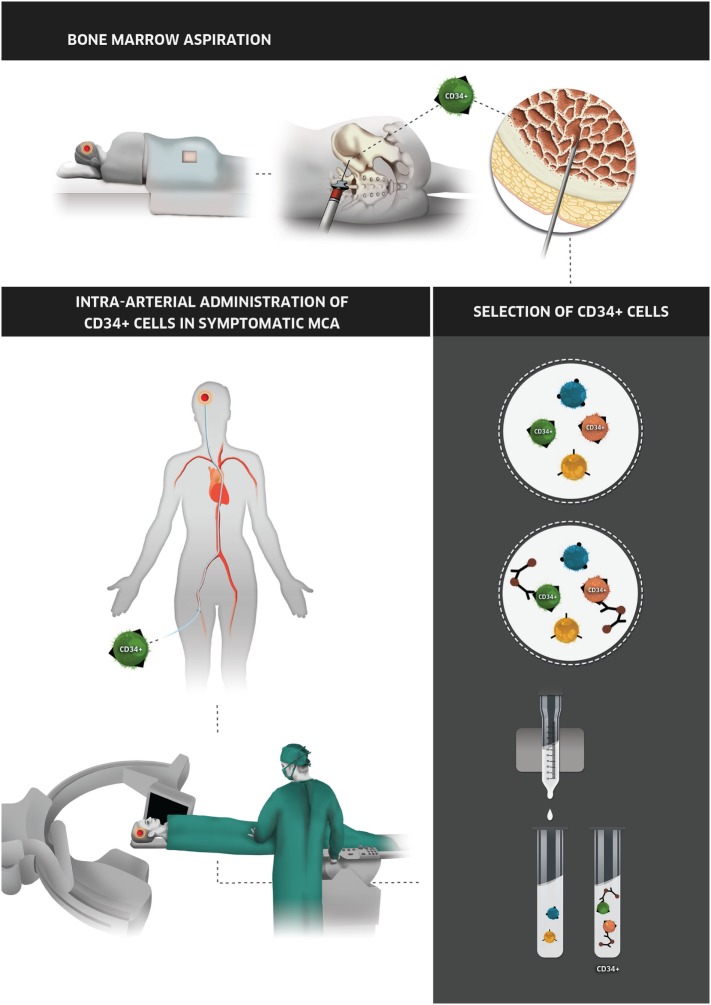
Illustrative representation of autotransplant of CD34+ cells. In the top of the image, bone marrow aspiration by hematologist is represented. The second step (bottom right) is the isolation of CD34+ cells, followed by the intra-arterial administration of the CD34+ in the symptomatic MCA (bottom left). Abbreviation: MCA, middle cerebral artery.

First, a single-pull 10-mL aspirate of bone marrow from the iliac crest will be collected by experienced Hematologist under aseptic conditions, using only local anesthesia at puncture site. CD34+ cells will then be isolated by magnetic cell sorting using CliniMACS Plus^®^ (Miltenyi Biotec) without cellular manipulation and suspended in 10–100 mL of saline solution. 24–48 h after collection, 1 × 10^6^ up to 1 × 10^8^ cells suspended in human albumin solution will be administered intra-arterially in the symptomatic MCA (Figure 2). Before infusion, all solutions will be screened for infections according to national human tissue transplantation requirements (national legislation 12/2009): Syphilis, HIV 1 and 2, Hepatitis B and C in all patients, as well as human T lymphotropic virus 1 and 2 whenever indicated.

In the angio-suite, catheterization of the symptomatic MCA by neuroradiologist will follow routine practice. In short, all patients will initially be submitted to local anesthesia of the groin for femoral artery puncture and intravenous administration of 5,000 units of unfractionated heparin. Then, a guide catheter selected according anatomical features of the patient will be navigated up to the initial segment of the symptomatic internal carotid artery. At that point, a microcatheter will navigate up to the beginning of the M1-MCA, where the solution containing the CD34+ cells will be slowly infused during approximately 10 min. The expected total duration of the procedure will be 30 min. All procedures will be accompanied by anesthesiologist. Sedation will not be used routinely, but will be possible according to the anesthesiologist’s choice. No other drug will be used routinely during the procedure, but the treating physicians will be free to use any other treatment considered necessary according to clinical condition. In case the interventionalist encounters a previously undiagnosed high-grade stenosis (>70%), vascular occlusion or any vascular malformation in the symptomatic arteries considered to be potentially hazardous in case of catheterization the treatment will be aborted.

Patients in the sham group will initially be subject to local anesthesia of the iliac crest by Hematologist (without bone marrow aspiration) and brought to the angio-suite 24–48 h later. All study participants will be blindfolded during the angiographic procedure and submitted to local anesthesia of the groin. Patients in the sham group will not be subject to arterial puncture and will remain in the angio-suite for approximately 30 min simulating cellular infusion. No information about treatment allocation will be transferred to the recovery staff at stroke unit. Subsequent medical caregivers will also be blinded to treatment allocation. The study physicians present during the angiographic procedure will have no further contact with the patient during the study.

Until clinical stabilization, the patients will remain in the hospital and perform a control magnetic resonance imaging (MRI) 48 h after treatment, followed by orientation by a dedicated rehabilitation center.

### Allocation Concealment

Stroke unit staff, patients, and outcome assessors are blinded to treatment allocation for the duration of the study. Unblinding will only take place whenever the treating physician decides that clinical management will critically depend on knowledge of treating allocation. Unblinding will be monitored and audited.

### Outcomes Assessed

The primary outcome will be ischemic stroke volume, measured by automated planimetric method on FLAIR imaging sequences of MRI performed 3 months after index event. The secondary outcomes are presented in Table 2. Neuroimaging assessment will include analysis of the three MRI performed during the study: before treatment, 48 h after treatment, and at 3 months. The neuroradiologist that will evaluate lesion volumes and physicians that will evaluate the secondary outcomes will not be involved in the initial management of the patient nor intervention and will be blinded for treatment allocation.

**Table 2:** Study outcomes assessed.

Primary outcome
Ischemic stroke volume on magnetic resonance imaging (MRI) performed at 3 months

**Secondary outcomes**

Hemorrhagic transformation (defined as type 2 parenchymal hemorrhage) Evolution of ischemic stroke volume from pre-treatment MRI to control MRI (48 h after intervention). Any stroke (ischemic stroke, intracerebral hemorrhage, and subarachnoid hemorrahge) Death of any cause Functional outcome at 3 months: mRS Impact of stroke throughout the first 3 months: Stroke Impact Scale Cognitive performance at 3 months: Montreal Cognitive Assessment scale (MoCA) Functional Independence at 3 months: Barthel Scale Upper limb Capacity at 3 months: Stroke Upper Limb Capacity Scale Mood at 3 months: Hospital Anxiety and Depression Scale Quality of life at 3 months: EuroQol Scale (EQ-5D) Gait speed at 3 months (10 m test) Temporo-spatial gait organization at 3 months (GAITRIte speed) Deglutition at 3 months: Functional Oral Intake Scale Language: Aphasia Rapid Test

### Data Monitoring Board

An independent Data and Safety and Monitoring Board (DSMB) will monitor any serious adverse events. It will be constituted by Neurologists and Internal Medicine physicians not involved in any part of the study design or execution. In the event of any safety concern, namely, rate of hemorrhagic transformation, cancer, procedural complications, or any other unexpected serious adverse event regarding the intervention group, the DSMB will make a recommendation to continue, stop, or modify the trial.

### Ethics

A signed written informed consent form will be sought by co-investigator stroke physicians and mandatory for participation in the trial. In the form all study procedures, potential risks and benefits are explained in simple terms. The study physicians will be responsible to explain and make sure the protocol is correctly understood by participants. All study subjects will be free to withdraw consent at any time. In case the patient is handicapped and not able to write down the required information, a legally acceptable representative will be allowed to fill out the informed consent form. Participants will be given a study number, which will be kept throughout all the analysis, maintaining confidentiality. All study participants will have a prespecified insurance to cover any adverse event of study participation.

Possible safety concerns rely on MRI acquisition, bone marrow aspiration, and angiographic catheterization (that will all follow routine protocols), as well as the autotransplant of CD34+ (that has been suggested to be safe in a phase I trial). Clinical follow-up after study completion will be the discretion of the treating physician.

An additional ethical concern will be to maintain blinding of the participants throughout the trial. Apart from the above mentioned design, all study and hospital staff personnel will receive training specific to this trial.

The study will be conducted in accordance with the principles of Good Clinical Practice, the Medical Research Involving Human Subjects act and the Declaration of Helsinki. The study has been approved by the funding body (COMPETE 2020, Ref: 3386). All protocol changes such as modifications in eligibility criteria, outcome measures, analyses, or study procedures will be resubmitted.

### Sample Size Estimates

No human randomized trials are available for effect estimate on intra-arterial administration of CD34+ for acute ischemic stroke. Moreover, animal studies are not ideal considering the specificity and inherent heterogeneity of human subjects. As such, for sample size calculation, we have used data from human observational studies on EPCs (7, 13). These studies indicated a large effect of EPCs in final ischemic lesion volume, with reported treatment effect sizes ranging from 1.2 to 2.0 (7, 13). For our sample size calculation of a unicenter pivotal phase IIa trial, we used an effect size of 1.2 for 90% power and a significance level of 5% to identify a difference between independent means of final infarct volume. A total of 30 patients was estimated for the trial: 10 patients treated at day 7 ± 2, 10 at day 20 ± 5, and 10 in the control group (5 sham procedures at each timepoint to ensure blinding).

### Expected Results

The authors expect that the autotransplant of CD34+ will be associated with lower infarct volumes at 3 months. Observational and *in vitro* data do not allow a rational expectation regarding the optimal time window for CD34+ administration (7 ± 2 vs. 20 ± 5 days). Considering the validated cellular protocol using autotransplantation and angiographic delivery using routine catheterization techniques, we expect to confirm the overall safety of this procedure, with no serious adverse events.

### Dissemination and Data Availability

The results will be disseminated through peer-reviewed publications, presentation at relevant scientific conferences and the general public.

The datasets generated during the study will be available on reasonable request.

### Study Organization and Funding

STROKE34 is part of StrokeTherapy (POCI-01-0247-FEDER-003386), executed by Stemlab, S.A. in Co-Promotion with Centro de Reabilitação do Centro - Rovisco Pais and Universidade de Coimbra, in partnership with Centro Hospitalar e Universitário de Coimbra. The trial is funded by the Operational Program Portugal 2020 and the European Regional Development Fund through COMPETE 2020.

## Discussion

The potential integration of stem cells in the treatment algorithm of acute ischemic stroke is still limited by several uncertainties, with most data coming from observational studies. STROKE34 is a randomized trial comparing different timepoints of CD34+ autotransplant: subacute (7 days) and late subacute stages (20 days). CD34+ have been confirmed to have a contributing role in the development of angiogenesis *in vivo* and *in vitro*. Angiogenesis plays a pivotal role in poststroke recovery; however, animal models have raised the possibility of hemorrhagic transformation as a potential complication of promoting new, immature vessels (14). Nonetheless, this link shows an apparent time-dependent response with acute disruption of the blood–brain barrier and hemorrhagic transformation in the first hours after stroke, whereas enhancing effective neoangiogenesis with improved outcomes in the subacute stage (8, 15). These facts reinforce the need to define an optimized therapeutic window for the design of future phase III trials.

Three methods are potential candidates for delivery of CD34+ cells: surgical cranial implantation, intravenous, and intra-arterial. Considering the invasiveness and potential hazards of surgical implantation as well as the significant absorption by non-target organs with intravenous delivery, intra-arterial administration seems to be the most promising candidate. This method is further supported by the increasing use of intra-arterial access for the treatment of acute stroke, and the safety and feasibility demonstrated in phase I study (10).

Another critical point in the design of this trial is the choice of therapeutic time window. Angiogenesis after stroke has been demonstrated from 3 to 4 days up to 5 weeks after injury (16–18). However, it is still unknown when CD34+ transplantation would be clinically effective. Early administration would imply exposing the highly active and metabolically demanding CD34+ cells to an inhospitable ischemic environment and would potentially promote ineffective angiogenesis and hemorrhagic transformation. Moreover, within the first 24 h, bone marrow aspiration would be contra-indicated in patients submitted to intravenous thrombolysis. Nonetheless, it is uncertain if later time windows would still have any clinical meaning, and the need for a permeable blood–brain barrier will ultimately limit cell delivery beyond the first 5 weeks (19). Altogether, a wide time frame is still potentially usable (from the first days up to 5 weeks) with presumably different biological effects and clinical implications. In this trial, we hope to answer if an earlier time window (5–9 days) is preferable compared to a later window (15–25 days).

A potential limitation of this study design is the small sample size planned. However, we have defined the primary endpoint as a neuroimaging variable (3 months infarct volume), to allow the inclusion of a smaller number of patients in this exploratory phase IIa trial, and have included comprehensive functional assessments as secondary outcomes.

## Ethics Statement

A signed written informed consent form will be sought by co-investigator stroke physicians and mandatory for participation in the trial. In the form all study procedures, potential risks and benefits are explained in simple terms. The study physicians will be responsible to explain and make sure the protocol is correctly understood by participants. In case the patient is handicapped and not able to write down the required information, a legally acceptable representative will be allowed to fill out the informed consent form. The study will be conducted in accordance with the principles of Good Clinical Practice, the Medical Research Involving Human Subjects act and the Declaration of Helsinki.

## Author Contributions

JS-F wrote the article. JS-F, LF, FS, GS, and LC defined the inclusion criteria and clinical design. LF, AG, TM, and CC defined the therapeutic product. CN and OG defined the neuroimaging protocol. JC defined the hematological protocol. AP, PA, JB, and VL defined the rehabilitation program and functional assessments.

## Conflict of Interest Statement

The authors declare that this study protocol was designed in the absence of any commercial or financial relationships that could be construed as a potential conflict of interest.
